# Association between monocyte Fcγ subclass expression and acute coronary syndrome

**DOI:** 10.1186/1742-4933-1-4

**Published:** 2004-11-12

**Authors:** David C Calverley, Taya Varteresian, Elizabeth Brass, Denice D Tsao-Wei, Susan Groshen, Wendy J Mack, Thomas A Buchanan, Howard N Hodis, Alan D Schreiber

**Affiliations:** 1 Department of Medicine, University of Colorado, Denver, CO; USA; 2 Department of Medicine, University of Southern California Keck School of Medicine, Los Angeles, CA; USA; 3 Department of Preventive Medicine, University of Southern California Keck School of Medicine, Los Angeles, CA; USA; 4 Department of Medicine, University of Pennsylvania School of Medicine, Philadelphia, PA; USA

## Abstract

**Background:**

Atherosclerosis lesions contain abundant immunoglobulins complexed with oxidized LDL (OxLDL) that are endocytosed by macrophages to form foam cells. While recent evidence supports a role for the macrophage scavenger receptor pathway in 75–90% of OxLDL uptake, in vitro evidence suggests another potential uptake pathway could involve autoantibody binding to IgG subclass-specific Fc receptors.

**Objective and Methods:**

To address this mechanism from an in vivo standpoint, the objective of this study was to utilize flow cytometry to prospectively determine monocyte Fcγ (FcR) I, II, and III receptor expression levels in patients with acute coronary syndrome (ACS, n = 48), diabetes mellitus (DM, n = 59), or neither (C, n = 88).

**Results:**

Increased FcR I expression was found in the ACS versus DM groups [geometric mean, (95% CI) = 2.26 (2.07, 2.47) versus 1.83 (1.69, 1.98) (p < 0.001)] and versus C [1.90 (1.78, 2.03) (p = 0.005)]. Similar relationships were found with both the FcR II receptor [ACS mean = 4.57 (4.02, 5.19) versus DM 3.61 (3.22, 4.05) (p = 0.021) and versus C 3.86 (3.51, 4.24) (p = 0.09)] and FcR III receptor [ACS mean = 1.55 (1.44, 1.68) versus DM 1.36 (1.27, 1.46) (p = 0.038) and versus C 1.37 (1.30, 1.45) (p = 0.032)]. There was no difference between DM and C groups in FcR I, II or III expression.

**Conclusions:**

This in vivo data supports a possible second OxLDL-autoantibody macrophage uptake mechanism through an Fc receptor-mediated pathway and a potential relationship between atherosclerotic plaque macrophage FcR levels and ACS.

## Introduction

Atherosclerosis is a chronic inflammatory process that results from hyperlipidemia and complex interactions involving other genetic and environmental factors. OxLDL plays a central role in the atherogenic process through generation of highly immunogenic neodeterminants for the immune system [1]. Natural autoantibody titer to a number of these epitopes and extent of immune complex formation may correlate with plaque size and rate of progression, and plaques have been shown to contain OxLDL/autoantibody immune complexes [2-5]. It is clear that both innate and adaptive immunity can modulate lesion progression and composition, and most studies to date have indicated a proatherogenic influence of the immune system on this process [1,4].

Recent evidence supports the macrophage scavenger receptors SR-A and CD36 as a mechanism responsible for up to 90% of uptake of OxLDL that leads to foam cell formation with no other scavenger receptors compensating for their absence in a knockout mouse model [6]. Earlier evidence involving in vitro incubation of both human monocyte-derived macrophages and the monocytic cell line THP-1 with human LDL-rabbit anti-apo B immune complexes demonstrated a potential role for the FcγRI receptor in its uptake [7]. A second in vitro study also suggested a potential Fc receptor role through inhibition of immune complex uptake when Fab or F(ab')_2 _fragments were substituted for an intact anti-apo B antibody [8]. Though findings from the latter two studies may have been partly explained by contributions from the scavenger pathway, it is reasonable to speculate the Fc receptor pathway maybe playing a small but important role as well [9-11].

Immune complexes with modified lipoproteins have recently emerged as an important coronary artery and macrovascular disease risk factor in DM [12,13]. Evidence supports an increased content of macrophages in the atherosclerotic lesions of persons with DM that is thought to be due to altered levels of cytokines [12]. Furthermore, while DM itself does not increase levels of LDL, the small dense LDL particles found in type 2 DM are more atherogenic because they are more easily glycated and are thought to be more susceptible to oxidation [14,15].

In recent work our group has shown FcγRII expression to be increased in the platelets of patients experiencing an acute atherothrombotic event, or who are healthy with two or more atherosclerosis risk factors [16]. Non-acutely ill diabetes patients have significantly elevated expression levels and this may play a role in the increased sensitivity of their platelets to activation by subendothelial collagen [16-19]. We speculate that Fc expression levels and activity on macrophages and platelets may represent another link between the immune system and atherosclerosis progression and plaque disruption.

In view of the controversy regarding the mechanism of cholesterol uptake by monocyte-macrophages in atherosclerosis and diabetes [20,21] and the previous lack of in vivo data to help elucidate any role of the Fc receptors in this process, we have prospectively determined IgG-binding receptor expression levels for each Fcγ receptor subclass on the monocytes of three groups: (1) patients admitted to the hospital with ACS, (2) well patients with no history of heart disease but one or more atherosclerosis risk factors (ARF's) that included DM, and (3) control patients (with no history of ACS or DM).

## Materials and Methods

All 195 patients were randomly chosen for study participation from a larger group who fit study inclusion criteria and gave written informed consent. Forty-eight patients in the study had heart disease (HD) and were within 2 hours of onset of an ACS (myocardial infarction or unstable angina), 59 were DM outpatients (both type 1 and type 2 were included) with no known history of HD, and an additional 88 outpatients without HD or DM were randomly chosen as controls (C). The number and nature of ARF's was documented for each group (Table 1).

**Table 1:** Demographic characteristics at enrollment

Characteristic	Group 1 (ACS)	Group 2 (DM)	Group 3 (C)	p-value^1^
Total patients (% with MI Group 1)	48 (52)	59	88	
Mean age (years)	56	55	56	0.86^2^
Male (%)	35 (73)	24 (41)	55 (63)	0.002
Positive family history (%)^3^	6 (13)	21 (36)	21 (24)	0.018
Current cigarette smoker (%)	16 (33)	10 (17)	5 (6)	<0.001
Hypertension (%)	29 (60)	29 (49)	20 (23)	<0.001
Abnormal lipid profile present (%)	24 (50)	12 (21)	17 (19)	<0.001
Diabetes mellitus present (%)	15 (31)	59 (100)	0 (0)	<0.001

1. Based on chi-square test.

2. Based on ANOVA F-test

3. One patient in group 2 had missing data with positive family history

(Abbreviations: ACS: acute coronary syndrome; DM: diabetes mellitus; C: control; MI: myocardial infarction)

Blood was collected in 3.8% trisodium citrate and divided into 50 μl aliquots to which 5 μl of a saturating concentration of anti-FcR I (32.2), anti-FcR II (IV.3), anti-FcR III (3G8), or a negative class-specific control antibody (MOPC-141, Sigma) was added. Following a 15 minute incubation, 5 μl of FITC-sheep anti-mouse antibody (Sigma) was added and a second 15 minute incubation done. The pellet was washed twice before 5 μl of phycoerythrin-conjugated anti-CD14 (Becton Dickinson) was added and incubated for 15 minutes. The solution was diluted with 1 ml ammonium chloride lysing solution and incubated for 10 minutes or until the solution was clear, and the pellet washed twice before flow cytometry to determine relative receptor expression levels was carried out according to the manufacturer's specifications (Becton Dickinson). Monocytes were readily identifiable from other blood cells by their forward and side scatter properties along with CD14 expression.

Following establishment of saturating concentrations for each antibody, mean inter and intra-individual coefficients of variation for each of the three Fc receptors were calculated in the antibody labeling assay employing blood samples from five healthy laboratory volunteers who met control patient criteria and FcR I values found to be 3.2 and 9.7%, FcR II 11.7 and 16.1% and FcR III 4.1 and 26.9% respectively.

The ratios of FcR I, FcR II, FcR III and MOPC-141 antibody expression were calculated for each patient and the logarithm of the ratios were used to analyze the results. An analysis of variance (ANOVA) was performed to compare the groups of (1) these 3 ratios with the 3 groups of patients, (2) the 3 ratios with the total number of major ARFs, (3) the 3 ratios with each ARF, such as hypertension, diabetes, and smoking, and (4) the 3 ratios with MI, and unstable angina in group 1. The overall p-values were based on the ANOVA F-test. If the overall F-test p-value < 0.05, the LSD method (least significant difference) [22] was used for multiple comparison. The geometric means and the associated 95% confidence intervals were calculated to summarize the data. Patient baseline data between groups was analyzed using the chi-square test. To perform statistical analysis, SAS software, version 8.2, was used (SAS Institute, Inc., Cary, NC).

## Results

**Figure 1:**
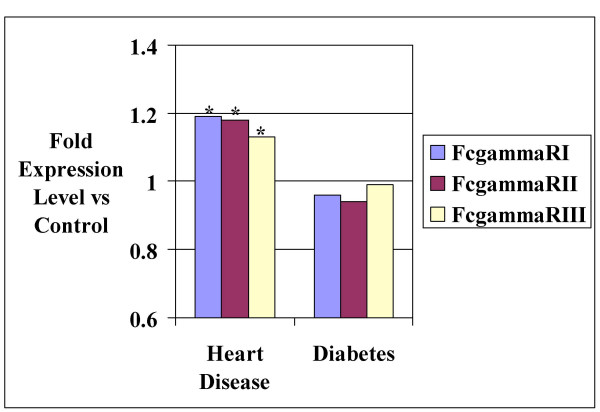
Monocyte Fcγ receptor subclass expression levels of 48 patients with heart disease (HD) and 59 patients with DM compared with 88 control patients with neither HD nor DM. HD patients display significantly increased expression levels of all 3 subclasses versus controls. * p = 0.002, 0.037, and 0.014 for FcR I, FcR II, and FcR III respectively when HD group is compared to control group.

Significantly increased FcR I expression was found in ACS patients compared with DM patients [geometric mean FcR I expression, (95% CI) = 2.26 (2.07, 2.47) versus 1.83 (1.69, 1.98) (p < 0.001)] and compared with C [1.90 (1.78, 2.03) (p = 0.005)] (Table 2, Figure 1). Similar relationships between the three groups were found to exist employing antibodies specific to the FcR II receptor: ACS geometric mean (CI) = 4.57 (4.02, 5.19) versus DM 3.61 (3.22, 4.05) (p = 0.021) and versus C 3.86 (3.51, 4.24) (p = 0.09) and the FcR III receptor: ACS geometric mean (CI) = 1.55 (1.44, 1.68) versus DM 1.36 (1.27, 1.46) (p = 0.038) and versus C 1.37 (1.30, 1.45) (p = 0.032). There was no difference between DM and C groups in FcR I, II or III expression (p = 0.73, 0.66, and 0.99 respectively).

**Table 2:** Mean monocyte FcR expression in 195 study patients

		FcR I		FcR II		FcR III	
	N	Geometric mean (95% CI)	p-value	Geometric mean (95% CI)	p-value	Geometric mean (95% CI)	p-value

Unadjusted analysis							
Groups			<0.001^2^		0.024^2^		0.021^2^
Group 1 (ACS)	48	2.26 (2.07, 2.47)	group 1 vs. 2: 0.001^3^	4.57 (4.02, 5.19)	group 1 vs. 2: 0.021^3^	1.55 (1.44,1.68)	group 1 vs. 2: 0.038^3^
Group 2 (DM)	59	1.83 (1.69, 1.98)	group 1 vs. 3: 0.005^3^	3.61 (3.22, 4.05)	group 1 vs. 3: 0.09^3^	1.36 (1.27, 1.46)	group 1 vs. 3: 0.032^3^
Group 3 (C)	88	1.90 (1.78, 2.03)	group 2 vs. 3: 0.73^3^	3.86 (3.51, 4.24)	group 2 vs. 3: 0.66^3^	1.37 (1.30, 1.45)	group 2 vs. 3: 0.99
Adjusted analysis^1^							
Groups			0.010^2^		0.061^2^		0.10^2^
Group 1 (ACS)	48	2.33 (2.14, 2.55)	group 1 vs. 2: 0.007^3^	4.57 (4.00, 5.22)	group 1 vs. 2: 0.050^3^	1.58 (1.45,1.71)	group 1 vs. 2: 0.10^3^
Group 2 (DM)	59	1.95 (1.78, 2.13)	group 1 vs. 3: 0.09^3^	3.71 (3.24, 4.24)	group 1 vs. 3: 0.47^3^	1.41 (1.30, 1.53)	group 1 vs. 3: 0.22^3^
Group 3 (C)	88	2.05 (1.87, 2.25)	group 2 vs. 3: 0.56^3^	4.11 (3.59, 4.71)	group 2 vs. 3: 0.38^3^	1.44 (1.32, 1.56)	group 2 vs. 3: 0.43^3^

1. Adjusted by smoking and hypertension status

2. p-value based on F-test

3. p-value based on Tukey-Kramer test

There were no statistically significant associations with increased expression of any Fc receptor and gender, family history of premature coronary disease, diabetes, or abnormal lipid profiles. Current cigarette smoking significantly increased expression of FcR I, 2.24 (2.01, 2.50), compared to absence of current cigarette smoking, 1.91 (1.82, 2.01) (p = 0.010) (Table 2 adjusted analysis). FcR II was significantly increased among the patients with hypertension, 4.29 (3.88, 4.74) compared to those without hypertension 3.73 (3.44, 4.05) (p = 0.034). There was a slight association between age and FcR I, II or III expression (p = 0.042, 0.050, 0.022 respectively). Expression levels in younger (age < 45) and older (age > 55) groups were higher than the middle age group. No difference was found in FcR expression with respect to ARF number (with the lone exception of increased FcR III in patients with 2 or more ARFs compared with less than two), or between the ACS subgroups of acute MI and unstable angina. When FcR expression is compared between all diabetes and non-diabetes patients in the 3 groups, there is no difference in monocyte FcR I, II, or III expression.

## Discussion

It can be speculated from this in vivo data that phagocytosis of OxLDL-autoantibody immune complexes by plaque-associated macrophage through an Fc-mediated pathway could be a second uptake mechanism in addition to that involving the scavenger receptors. The potential clinical implication behind these findings is that while marrow and blood monocyte scavenger receptors SR-A and CD36 have not demonstrated inter-individual variability in their basal expression levels (prior to initial uptake of OxLDL or differentiation to macrophages) [23,24], the variable expression of Fcγ receptors found in this series of patients maybe playing a role in the extent of OxLDL immune complex uptake by atherosclerosis plaques. The fact we were able to document relatively increased surface expression of all three receptor classes in patients with ACS, along with increased FcR I in those who smoked and FcR II in those with hypertension, supports this hypothesis. Any precise pathophysiological implication behind these findings, though, or any cause and effect relationship between monocyte Fc expression and ACS is presently uncertain.

No difference was noted in expression of any Fc receptor between the diabetes and control groups. Given that the control group was the largest of the three and that there was a difference noted in the expression levels of all three receptors between the two smaller groups, it can be concluded that increasing the sample size of either of the two groups with similar expression levels could possibly lead to an increase in the difference between them, but this difference is still unlikely to be of any significance compared with that between the ACS group and the other two groups.

There was an interesting trend in both the unadjusted and adjusted analyses (Table 2) in which the control groups had consistently higher monocyte Fc expression levels compared with the diabetes groups. One potential explanation for this would be the hypothesis that those with diabetes may have reduced FcR expression levels (with a possible consequent decreased uptake in oxidized LDL) compared with non-diabetes subjects in response to relatively higher levels of molecular mediators that support atherosclerosis progression and a pro-inflammatory, pro-thrombotic environment. This may reflect a biological ying-yang type of response that leads to an attempt at dampening the effects of molecular players capable of contributing to atherothrombotic events.

An issue pertinent to this study would be the possible effects of inflammation on monocyte FcR expression levels. In diabetes patients it would be reasonable to speculate a significant number of activated cells in the circulation would be unlikely since in most patients with atherosclerosis the inflammatory reaction is circumscribed to the vessel wall. Overexpression of monocyte Fc receptors may have been a possibility in ACS however. Figure 1 shows the ratio of ACS/control mean FcR expression levels to be very similar between Fc receptor subtypes. The extent of increased expression associated with inflammation-associated monocyte activation has been shown to be variable between FcR subtypes [25]. In this respect FcR II and III represent the receptors primarily involved in the inflammatory response in vivo. Since all three receptors had uniform increases in expression levels in ACS compared with controls (with the FcR I ratio being the highest of the three), it may be reasonable to attribute a relatively minimal effect of acute inflammation to the ACS data. Circulating monocyte activation may also have returned relatively close to baseline as a consequence of blood being drawn around 2 hours after symptom onset in most cases. The average circulation time of blood monocytes in response to an inflammatory stimulus may fall to as little as 30 minutes [26].

Observational studies of this nature have certain limitations related to their design and patient selection. As an example, selection bias needs to be considered in any study involving a population of volunteers associated with an atherosclerosis prevention trial (Groups 2 and 3). Even though the ethnic composition of the groups was the same, the implication of this selection bias is that the results are not generalizable to the population at large and this is attested to by the demographic characteristics in Table 1.

Through bridging innate and adaptive immune processes, macrophages play an important role in the progression of atherosclerosis and mediating plaque disruption that is considered to be the inciting event in the majority of coronary thrombi [27,28]. In this respect there is continual migration of monocytes between neighboring endothelial cells as well as two-way migration of monocytes between blood and OxLDL-containing foam cells when there is separation of endothelial cells associated with the fatty streak [29]. This observation and the inherent difficulty in isolating monocytes from tissues compared with serum both served as justification for utilizing blood monocyte Fc expression as a surrogate for plaque macrophage expression [30].

The overall effect of the humoral immune response on atherogenesis is likely to be complex. Of note, for example, is that the FcγRII receptor has an inhibitory role on B cells that are rarely seen in plaques, while it mediates phagocytosis and release of inflammatory mediators from cells of the myeloid lineage when cross-linked by immune complexes [31,32]. Thus FcR binding by opsonized OxLDL could induce either negative or positive regulation of immune cell responses. Elucidation of the immune mechanisms involved in atherogenesis will continue to evolve and lead to new insights into the molecular pathways associated with disease progression. Ultimately these insights will contribute towards the full explanation behind the clinical diversity of atherosclerosis expression in patients who appear to have equal risk.

## Competing Interests

The authors declare that they have no competing interests.

## References

[B1] Binder CJ, Chang M.-K, Shaw PX, Miller Y, Vartvigsen K, Dewan A, Witztum JL (2002). Innate and acquired immunity in atherogenesis. Nature and Medicine.

[B2] Palinski W, Witztum JL (2000). Immune responses to oxidative neoepitopes on LDL and phospholipids modulate the development of atherosclerosis. J Int Med.

[B3] Witztum JL, Palinski W (1999). Are immunological mechanisms relevant for the development of atherosclerosis?. Clin Immunol.

[B4] Hansson GK (2001). Immune mechanisms in atherosclerosis. Arterioscl Thromb Vasc Biol.

[B5] Yla-Herttuala S, Palinski W, Butler SW, Picard S, Steinberg D, Witztum JL (1994). Rabbit and human atherosclerotic lesions contain IgG that recognizes epitopes of oxidized LDL. Arterioscler Thromb.

[B6] Kunjathoor VV, Febbraio M, Podrez EA, Moore KJ, Andersson L, Koehn S, Rhee JS, Silverstein R, Hoff HF, Freeman MW (2002). Scavenger receptors class A-I/II and CD36 are the principal receptors responsible for the uptake of modified low density lipoprotein leading to lipid loading in macrophages. J Biol Chem.

[B7] Lopes-Virella MF, Binzafar N, Rackley S, Takei A, La Via M, Virella G (1997). The uptake of LDL-IC by human macrophages: predominant involvement of the FcγRI receptor. Atherosclerosis.

[B8] Khoo JC, Miller E, Pio F, Steinberg D, Witztum JL (1992). Monoclonal antibodies against LDL further enhance macrophage uptake of LDL aggregates. Arterioscler Thromb.

[B9] Griffith RL, Virella GT, Stevenson HC, Lopes-Virella MF (1988). Low density lipoprotein metabolism by human macrophages activated with low density lipoprotein immune complexes. A possible mechanism of foam cell formation. J Exp Med.

[B10] Klimov AN, Denisenko AD, Popov AV, Nagornev VA, Pleskov VM, Vinogradov AG, Denisenko TV, Magracheva E, Kheifes GM, Kuznetzov AS (1985). Lipoprotein-antibody immune complexes: Their catabolism and role in foam cell formation. Atherosclerosis.

[B11] Gisinger C, Virella GT, Lopes-Virella MF (1991). Erythrocyte-bound low-density lipoprotein immune complexes lead to cholesteryl ester accumulation in human monocyte-derived macrophages. Clin Immunol Immunopath.

[B12] Eckel RH, Wassef M, Chait A, Sobel B, Barrett E, King G, Lopes-Virella M, Reusch J, Ruderman N, Steiner G, Vlassara H (2002). Prevention Conference VI: Diabetes and Cardiovascular Disease: Writing Group II: Pathogenesis of atherosclerosis in diabetes.

[B13] Lopes-Virella MF, Virella G, Orchard TJ, Koskinen S, Evans RW, Becker DJ, Forrest KY (1999). Antibodies to oxidized LDL and LDL-containing immune complexes as risk factors for coronary artery disease in diabetes mellitus. Clin Immunol.

[B14] Powers A (2003). Ch.333: Diabetes mellitus. Harrison's Online.

[B15] Semenkovich CF, Heinecke JW (1997). The mystery of diabetes and atherosclerosis: time for a new plot. Diabetes.

[B16] Calverley DC, Brass E, Hacker MR, Tsao-Wei DD, Espina BM, Pullarkat VA, Hodis HN, Groshen S (2002). Potential role of platelet FcγRIIA in collagen-mediated platelet activation associated with atherothrombosis. Atherosclerosis.

[B17] Calverley DC, Hacker MR, Loda KA, Brass E, Buchanan TA, Tsao-Wei DD, Groshen S (2003). Increased platelet Fc receptor expression as a potential contributing cause of platelet hypersensitivity to collagen in diabetes mellitus. Br J Haematol.

[B18] Watson SP, Asazuma N, Atkinson B, Berlanga D, Best D, Bobe R, Jarvis G, Marshall S, Snell D, Stafford M, Tulasne D, Wilde J, Wonerow P, Frampton J (2001). The role of ITAM- and ITIM-coupled receptors in platelet activation by collagen. Thromb Haemost.

[B19] Yanaga F, Poole A, Asselin J, Blake R, Schieven GL, Clark EA, Law CL, Watson SP (1995). Syk interacts with tyrosine-phosphorylated proteins in human platelets activated by collagen and cross-linking of the Fcγ-IIA receptor. Biochem J.

[B20] Lusis AJ (2000). Atherosclerosis. Nature.

[B21] Kruth HS (2001). Macrophage foam cells and atherosclerosis. Frontiers in Bioscience.

[B22] Snedecor GW, Cochran WG, Eds (1973). Statistical methods.

[B23] Hughes DA, Fraser IP, Gordon S (1995). Murine macrophage scavenger receptor: in vivo expression and function as receptor for macrophage adhesion in lymphoid and non-lymphoid organs. Eur J Immunol.

[B24] Peiser L, Gordon S (2001). The function of scavenger receptors expressed by macrophages and their role in the regulation of inflammation. Microbes and Infection.

[B25] Takai T, Li M, Sylvestre D, Clynes R, Ravetch JV (1994). FcRγ chain deletion results in pleiotrophic effector cell defects. Cell.

[B26] Meuret G, Hoffman G (1973). Monocyte kinetic studies in normal and disease states. Br J Haematol.

[B27] Falk E (1991). Coronary thrombosis: pathogenesis and clinical manifestations. Am J Cardiol.

[B28] Witztum JL (2002). Splenic immunity and atherosclerosis: a glimpse into a novel paradigm?. J Clin Invest.

[B29] Faggiotto A, Ross R, Harker L (1984). Studies of hypercholesterolemia in the nonhuman primate. I. Changes thst lead to fatty streak formation. Arteriosclerosis.

[B30] Tertov VV, Orekhov AN, Sayadyan KS, Serebrennikov SG, Kacharava AG, Lyakishev AA, Smirnov VN (1990). Correlation between cholesterol content in circulating immune complexes and atherogenic properties of CHD patients' serum manifested in cell culture. Atherosclerosis.

[B31] Uher F, Lamers MC, Dickler HB (1999). Antigen-antibody complexes bound to B-lymphocyte Fcγ receptors regulate B-lymphocyte differentiation. Cell Immunol.

[B32] Kurosaki T, Ravetch JV (1989). A single amino acid in the glycosyl phosphatidylinositol attachment domain determines the membrane topology of FcγRIII. Nature.

